# Erie County Medical Society

**Published:** 1856-12

**Authors:** 


*Erie County Medical Society.*—An alternative mandamus has been issued by Judge Marvin, of the Supreme Court, citing the Erie County Medical Society to appear at Angelica, Allegany County, and show cause why John D. Hill should not be restored to membership in the society. The application is based on alledged informality in the proceedings upon which Hill was expelled. The society met on Saturday, the 22d of Nov., to take action in the matter. The meeting was largely attended, and the determination to stand by their previous action was unanimous. No member present manifested the least hesitation as to the proper course to pursue. Resting on the justice of their original action, and determined to maintain its own rights and dignity at any expense, no man was found to express a word of sympathy, or a suggestion of lenity, for the plaintiff. It was at once decided to employ counsel and defeat the mandamus, if possible; and we are happy to be assured that there is little question that the society will be vindicated by the court. Should it not be, it still remains to be tested, whether or no the society shall be compelled to submit to the demands of every contumacious member. At no time in the history of this discussion, has the society been so much a unit. They have, by prudence and firmness, obtained large concessions from the county authorities, and a better feeling is rapidly taking the place of that uncharitableness, which began and was maintained by an insignificant faction, which having no merits of its own to rely upon, could only succeed by detraction of the merits of others. Our friends have now only to maintain their position for a little longer. The defeat of the opposition will be final and total; once subdued, they will not be in a position to do any further damage.

To return to the meeting. The President, Dr. Van Pelt; Vice-president, Dr. Hamilton; Secretary, Dr. Newman; Treasurer, Dr. Wilcox; and Dr. Hunt; were appointed a committee to employ counsel and act for the society,

Messrs. Talcott and Thompson, two distinguished legal gentlemen, have been retained, and the case will be argued on the 1st of December, 1856.

